# ExUTR: a novel pipeline for large-scale prediction of 3′-UTR sequences from NGS data

**DOI:** 10.1186/s12864-017-4241-1

**Published:** 2017-11-06

**Authors:** Zixia Huang, Emma C. Teeling

**Affiliations:** UCD School of Biology and Environmental Science, University College Dublin, Belfield, Dublin 4, Ireland

**Keywords:** 3′-UTR prediction, Next generation sequencing, Independent of genomes

## Abstract

**Background:**

The three prime untranslated region (3′-UTR) is known to play a pivotal role in modulating gene expression by determining the fate of mRNA. Many crucial developmental events, such as mammalian spermatogenesis, tissue patterning, sex determination and neurogenesis, rely heavily on post-transcriptional regulation by the 3′-UTR. However, 3′-UTR biology seems to be a relatively untapped field, with only limited tools and 3′-UTR resources available. To elucidate the regulatory mechanisms of the 3′-UTR on gene expression, firstly the 3′-UTR sequences must be identified. Current 3′-UTR mining tools, such as GETUTR, 3USS and UTRscan, all depend on a well-annotated reference genome or curated 3′-UTR sequences, which hinders their application on a myriad of non-model organisms where the genomes are not available. To address these issues, the establishment of an NGS-based, automated pipeline is urgently needed for genome-wide 3′-UTR prediction in the absence of reference genomes.

**Results:**

Here, we propose *ExUTR*, a novel NGS-based pipeline to predict and retrieve 3′-UTR sequences from RNA-Seq experiments, particularly designed for non-model species lacking well-annotated genomes. This pipeline integrates cutting-edge bioinformatics tools, databases (Uniprot and UTRdb) and novel in-house Perl scripts, implementing a fully automated workflow. By taking transcriptome assemblies as inputs, this pipeline identifies 3′-UTR signals based primarily on the intrinsic features of transcripts, and outputs predicted 3′-UTR candidates together with associated annotations. In addition, *ExUTR* only requires minimal computational resources, which facilitates its implementation on a standard desktop computer with reasonable runtime, making it affordable to use for most laboratories. We also demonstrate the functionality and extensibility of this pipeline using publically available RNA-Seq data from both model and non-model species, and further validate the accuracy of predicted 3′-UTR using both well-characterized 3′-UTR resources and 3P–Seq data.

**Conclusions:**

*ExUTR* is a practical and powerful workflow that enables rapid genome-wide 3′-UTR discovery from NGS data. The candidates predicted through this pipeline will further advance the study of miRNA target prediction, *cis* elements in 3′-UTR and the evolution and biology of 3′-UTRs. Being independent of a well-annotated reference genome will dramatically expand its application to much broader research area, encompassing all species for which RNA-Seq is available.

**Electronic supplementary material:**

The online version of this article (10.1186/s12864-017-4241-1) contains supplementary material, which is available to authorized users.

## Background

The three prime untranslated region (3′-UTR) is the regulatory noncoding section of an mRNA, which plays a crucial role in mediating temporal and spatial gene expression [1, 2]. Structurally, the 3′-UTR immediately follows the stop codon and terminates at the polyadenylation cleavage site of a transcript, where a variety of *cis* sequence elements are located including microRNA response elements (MREs), AU-rich elements (AREs) and polyadenylation signals (PASs) [1, 3]. These regulatory elements are recognized, and further interact with *trans* factors, which determine the fate of mRNA by influencing their stability, subcellular localization and translation efficiency. In eukaryotes, the 3′-UTR has considerable variation in number and length across taxa, with higher level organisms typically having more and longer 3′-UTRs than lower eukaryotes [3, 4]. In addition, certain isoforms which differ only in 3′-UTR can be generated through alternative polyadenylation during transcription, resulting in the expression of the same protein but in varying amounts and subcellular locations [1, 5]. These complex patterns of 3′-UTR regulation have been associated with morphological diversity, embryogenesis, tissue patterning and tumorigenesis [3, 4, 6, 7]. Therefore, insights into the regulatory mechanisms of how the 3′-UTR regulates gene expression will enable a better understanding of the molecular basis for evolution, morphology and developmental biology.

The past ten years has witnessed tremendous advancements in ‘Omic’ technologies, with high-throughput sequencing revolutionizing the field of molecular biology [8]. RNA-Seq, a next generation sequencing (NGS) method, enables the sequencing of RNA from any species at an unprecedented resolution and scale, providing novel means to tackle outstanding questions regarding the regulatory mechanisms of the 3′-UTR. For example, Mangone et al. [9] comprehensively defined the 3′-UTR landscape in *C. elegans* using Roche/454 pyrosequencing, and Xia et al. [10] studied the patterns of 3′-UTR alternative polyadenylation across seven tumor types in human using Illumina Sequencing. However, despite the large amount of RNA sequencing data generated from multiple taxa, 3′-UTR studies have typically been restricted to a few model organisms, whose well-annotated genomes were available. Few curated 3′-UTR resources and genome-wide methods are available to predict and extract 3′-UTR sequences from the wealth of NGS data now freely available, ultimately limiting comparative studies required to advance this field.

The UTRdb [11], Ensembl [12] and UCSC [13] are currently the most popular databases that curate 3′-UTR sequences. UTRdb contains a total of ~660 thousand entries across 110 species but has not been regularly updated, while Ensembl and UCSC provide well-assembled genomes, but many 3′-UTR regions remain poorly annotated. Regarding the tools for 3′-UTR prediction, 3USS [14] and UTRscan [11], both user-friendly web servers, were developed with the aim of retrieving 3′-UTR sequences from RNA transcripts. However, these tools rely strongly on reference genomes or current curated UTR databases and are therefore restricted to several well-studied species. Additionally, a recent well-designed standalone tool, GETUTR [15], employed heuristic and regression methods to precisely predict 3′-UTRs from large-scale RNA-Seq data. This approach not only demands intensive computational capacity but also requires high-coverage well-assembled genomes, with excellent annotation, therefore limiting its application for non-model organisms. To date, no pipelines have been developed and published for use on non-model organisms. These shortcomings limit the acquisition of 3′-UTR sequences from countless studies of non-model organisms, thus hindering a systematic exploration of the molecular mechanisms that underlie the effect of 3′-UTR on gene regulation. To address this problem, we have developed a novel pipeline *ExUTR*, which enables genome-wide identification of 3′-UTR without references and annotations from massive RNA-Seq experiments.

*ExUTR* uses a genome-wide approach for the prediction of a 3′-UTR landscape from RNA-Seq experiments, irrespective of the availability of well-assembled reference genomes. Using this pipeline, we successfully obtained a large number of 3′-UTR candidates from both reference-based and de novo transcriptome assemblies of model and non-model mammals. A large overlap was observed between the 3′-UTR candidates and the curated 3′-UTR resources, and analyses of 3P–Seq data and their corresponding RNA-Seq data indicate the accuracy and reliability of the *ExUTR* pipeline. More importantly, *ExUTR* outweighs other current methods as it can correctly predict 3′-UTRs from de novo assembled transcriptomes, even in the absence of well-annotated genomes of the relevant species. Therefore, *ExUTR* can be used in RNA-Seq transcriptomic studies across diverse tissue types, developmental stages and physiological conditions, for both model organisms and non-model species, enabling a wider phylogenomic perspective on 3′-UTR biology.

## Implementation

**Fig. 1:**
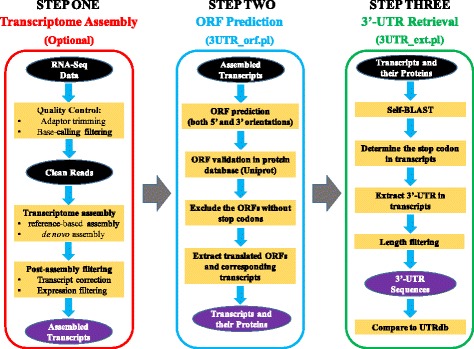
The workflow of the *ExUTR* pipeline. Transcriptome assembly (Step One) is optional if assembled transcripts are available. ORF prediction and 3′-UTR retrieval steps are implemented in the *UTR_orf.pl* and *UTR_ext.pl* scripts, respectively

*ExUTR* is a Linux-based pipeline implementing a fully integrative analysis workflow, designed to incorporate core in-house Perl scripts as well as free third-party software tools and databases. Standard input and output formats, such as FASTQ, FASTA and BLAST output format, are used to facilitate the modularity between different software packages, allowing users to run certain modules only as needed. Compared to existing tools that depend mainly on well-annotated genomes, the design of *ExUTR* enables the prediction of 3′-UTRs based solely on the intrinsic signals of assembled transcripts, in the absence of reference genomes and related annotations. The architecture of *ExUTR* contains three steps, consisting of **1)** Transcriptome assembly; **2)** ORF prediction; **3)** 3′-UTR sequence retrieval. Each step is described in detail below, and the workflow of the pipeline is visualized in Fig. 1. More details for usage are described at https://github.com/huangzixia/ExUTR.

### Step one: Transcriptome assembly

Since *ExUTR* predicts 3′-UTR sequences based mainly on the intrinsic signals of the transcripts, the acquisition of a robust transcriptome assembly is optimal. Therefore, full-length transcripts are required to be constructed from RNA-Seq data using either reference-based methods or de novo methods. This whole process can be simply achieved by orchestrating highly standardized third-party software packages (see below), which can flexibly meet diversified needs from users due to varied experimental designs. Here, we suggest a procedure to generate the high-quality transcriptome assembly for 3′-UTR prediction.

In general, prior to transcriptome assembly, adaptor sequences and low-quality regions are removed from raw FASTQ reads. This can be done by several popular quality-inspection tools such as Cutadapt [16], NGS QC toolkit [17] or Trimmomatic [18]. If a reference genome is available, the post-processed reads can be mapped against the genome using well-known spliced aligners such as Tophat2 [19], STAR [20] or HISAT [21], and then assembled into transcripts (isoforms) by Cufflinks [22]. When lacking a reference, a de novo assembly method can also be carried out using Trinity [23], SOAPdenovo [24] or other equivalent assemblers. The resulting assembled transcripts (FASTA file) could be theoretically imported into the next step for ORF prediction. However, it is strongly encouraged that their quality should be further screened due to the potential for misassembled transcripts, false positive alternatively spliced isoforms or artefacts. The misassembled transcripts with unexpected ‘indels’ within an ORF region can be corrected using FrameDP [25], while artefacts or false positive isoforms, which are usually expressed at extremely low levels, are deemed as unreliable, and should also be eliminated. Typically, assemblers, such as Cufflinks, can automatically report transcript abundances (FPKM) during assembly. Otherwise, transcripts could be quantified by alignment-based software like RSEM [26], or newly-developed tools which implement pseudo-alignment algorithms, such as Sailfish [27] or Salmon [28]. The post-assembly quality control is particularly important for de novo assemblers from which many misassembled or false positive transcripts are created.

The pipeline suggested in Step One allows users to easily customize the combination of third-party programs and their associated parameters to generate high-quality assemblies, due to varied demands. For the users who have already obtained robust assemblies, the whole step could be skipped by directly importing transcripts into Step Two for open reading frame (ORF) prediction and annotation.

### Step two: ORF prediction

Since the 3′-UTR immediately starts from the stop codon of a transcript, it is necessary that the structure of the sequence should be investigated, especially the open reading frame (ORF) which is crucial to determine the position of the 3′-UTR in a transcript. In *ExUTR*, we employ a self-predicting method to extrapolate the ORFs in transcripts. In brief, for unstranded RNA-Seq experiments, transcripts are factitiously translated into protein sequences in all six possible reading frames, three in each of forward and reverse directions. This is required as both forward and reverse complementary transcripts will be produced during assembly. As it is well justified that the longest reading frame is most likely to be used in translation [29], we select the longest predicted ORFs for each orientation, and further validate them by aligning them to the putative protein databases, such as Uniprot, using BLASTP [30]. For each transcript, we sort these two longest ORFs by length, and firstly validate the longer one. If the longer one has no BLAST hits, the shorter one will be used for similarity search. For stranded-specific RNA-Seq experiments, either three forward or reverse ORFs depending on the sequencing directions are investigated, the longest of which is further validated using Uniprot. This validation step ensures the authenticity of these ORFs. Using BioPerl modules [31], BLAST reports are subsequently parsed to annotate the ORFs through assigning the gene name of the best hit to the sequence. To avoid certain noncoding RNA that have limited ORF potential, such as LncRNA, being annotated as protein-coding transcripts, employing stringent BLAST parameters by setting E-value, alignment similarity and coverage is highly recommended. Sequences without BLAST hits are excluded from further analysis since they may represent spurious artefacts in the assembly, or transcripts with no potential ORFs. For the sake of determining the 3′-UTR in the sequence, ORFs that have no stop codons are also abandoned.

All these steps are implemented in the *3UTR_orf.pl* script (Fig. 1), which takes assembled transcripts in FASTA format as input, and outputs the annotated transcripts with potential ORF and unambiguous stop codon, and their corresponding amino acid sequences, both in FASTA format. The default parameter settings of the external programs are applied in the script, but can be readily modified to cater for users’ needs.

### Step three: 3′-UTR sequence retrieval

This step implements the key concept of *ExUTR*, that the prediction of 3′-UTR is based solely on transcript sequences without the requirement of reference genomes. To achieve this, the transcripts, which are exported from Step Two, are accordingly aligned with their predicted amino acid sequences using BLASTX [30]. This comparison potentially allows a transcript to have the best alignment with the amino acid sequence of its own, although multiple hits could be detected due to paralogs or isoforms. Therefore, the alignments only between transcripts and their corresponding amino acid sequences are parsed and analyzed, and the position of the stop codon will be subsequently calculated for each transcript. Note that the orientation of a transcript is considered when interpreting the alignments, so that the position of the stop codon could be accordingly marked in the sequence. Once obtaining the location of the stop codon, the 3′-UTR should be simply retrieved from a transcript by trimming all regions before the stop codon, including the ORF, possibly 5′-UTR and the stop codon itself. These predicted 3′-UTR sequences will be further filtered by length, which could be readily modified by users. The validation of these resulting 3′-UTR candidates is conducted by aligning them to the UTRdb database or other curated 3′-UTR resources using BLASTN [30], and the result will be further summarized.

All these steps regarding 3′-UTR prediction, sequence retrieval and validation are carried out by the *3UTR_ext.pl* script. It takes a set of transcripts and their predicted amino acid sequences as inputs, and outputs 3′-UTR sequences (FASTA file) together with a tab-delimited, CSV-compatible sheet containing 3′-UTR annotation.

## Results

### 3′-UTR prediction: Case studies using *ExUTR*

To demonstrate the functionality and extensibility of *ExUTR*, we employed publically available RNA-Seq data from both model and non-model mammals (Table 1). For each model species, we assembled the transcriptome using both reference-based and de novo methods while for non-model species only de novo assembly was performed. Details on the procedures to obtain the assembled transcripts (Step One) are extensively described in Additional file 1. The de novo assembly step was implemented on a computational cluster, while all other procedures were performed on an Ubuntu-based desktop equipped with 8 CPU Intel Core-i7 Processor and 16G memory.

**Table 1:** Summary of RNA-Seq data tested on *ExUTR*

Species	Tissue	RNA-Seq library	Assembly method	No. of assembled transcripts	*ExUTR* runtime
Bat (*Myotis myotis*)	Blood	SRX763357	reference-based	22,265	2 h 16 min
			de novo	34,913	2 h 25 min
Cow (*Bos taurus*)	Brain	SRX764721	reference-based	40,904	4 h 36 min
			de novo	93,137	3 h 57 min
Mouse (*Mus musculus*)	Kidney	SRX1603138	reference-based	53,892	5 h 45 min
			de novo	125,643	6 h 02 min
Pig (*Sus scrofa*)	Blood	SRX242932	reference-based	32,431	2 h 54 min
			de novo	28,540	0 h 51 min
Rat (*Rattus norvegicus*)	Brain	SRX471401	reference-based	82,558	4 h 59 min
			de novo	69,293	1 h 33 min
Human (*Home sapien*s)	Liver	ERX1217498	reference-based	186,053	10 h 44 min
			de novo	713,017	10 h 49 min
Jamaican fruit bat (*Artibeus jamaicensis*)	Spleen	SRX176203	de novo	225,089	6 h 36 min
Arctic fox (*Vulpes lagopus*)	Mixed	ERX632794	de novo	147,593	6 h 21 min
Spiny mouse (*Acomys cahirinus*)	Brain	SRX1818436	de novo	203,621	5 h 57 min
Long-haired mouse (*Abrothrix hirtus*)	Kidney	SRX663111	de novo	101,036	4 h 38 min
Grey wolf (*Canis lupus*)	Blood	SRX1713277	de novo	100,501	5 h 19 min
Sika deer (*Cervus nippon*)	Antler	ERX024230 ERX028792	de novo	56,737	3 h 26 min

**Fig. 2:**
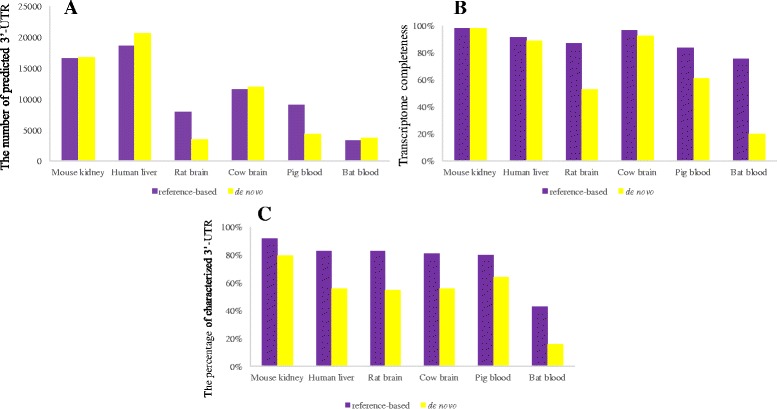
The statistics of 3′-UTR prediction through the *ExUTR* pipeline. **a** The number of 3′-UTR candidates predicted from the reference-based and de novo assemblies from six model species. **b** The transcriptome completeness evaluated by CEGMA. **c** The percentages of 3′-UTR candidates that have been characterized in their respective genomes

**Fig. 3:**
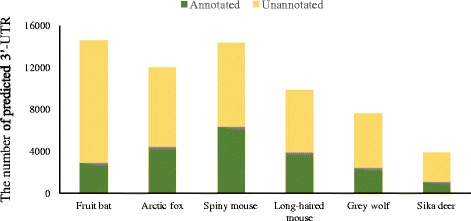
The number of 3′-UTRs predicted from six non-model mammals using *ExUTR*. The green color indicates the number of 3′-UTRs that had homologs in the UTRdb database

Using *ExUTR*, a genome-wide scale of 3′-UTR was predicted from both reference-based and de novo assemblies, although the numbers varied among different species (Fig. 2a). This is because the number of predicted 3′-UTRs is highly associated with completeness of the transcriptome owing to sequencing depth, assembly quality, as well as species, tissue types and experimental conditions. We assessed the transcriptome completeness for each assembly (See Additional file 1 for the method), and a strong correlation was found between the number of predicted 3′-UTR and the levels of transcriptome completeness (Reference-based r^2^ = 0.805; de novo r^2^ = 0.814; Spearman’s rank correlation tests). Therefore, a higher number of 3′-UTRs were expected from human, cow and mouse transcriptomes (Fig. 2a and b). Notably, a large proportion of 3′-UTR candidates have been characterized in their respective genomes, with the range of 43.3% ~ 91.7% (average 76.9%) for reference-based assemblies while 16% ~ 84.2% (average 60.8%) for de novo assemblies (Fig. 2c). The large overlap convincingly illustrates the accuracy and functionality of the pipeline. In particular, all species except bat showed that at least 80% of predicted 3′-UTRs were characterized when using the reference-based strategy (Fig. 2c). In addition to these model species whose genomic sequences have been comprehensively studied and well curated, a large number of 3′-UTR candidates were also predicted from non-model mammals using *ExUTR* (Fig. 3). However, the average percentage of 3′-UTRs that have been characterized was only 33.5%, significantly lower than that of model organisms (*P* < 0.05, Wilcoxon signed rank test). However, attention must also be paid to those candidates that have not been well characterized, in that they are likely to be potential novel 3′-UTR. It is commonly accepted that the 3′-UTR sequences, many of which are regarded as species-specific, evolve much faster than protein coding regions and have not been fully recorded yet [32]. Thus, further confirmation by PCR and Sanger sequencing is needed.

**Fig. 4:**
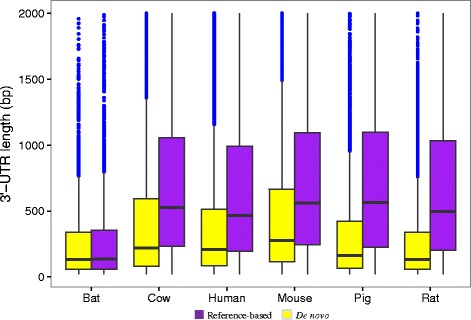
Length distributions of 3′-UTR candidates predicted from the reference-based and de novo assemblies of six model mammals

Completeness of the 3′-UTR is essential to investigate their regulatory mechanisms. To evaluate the lengths of 3′-UTRs derived from both reference-based and de novo assemblies, we compared their distributions for each species. Apart from bat, the length distributions obtained from the reference-based assemblies are significantly longer than those predicted from the de novo assemblies (*P* < 0.01) (Fig. 4). This phenomenon could reasonably be explained by the quality of transcripts that relatively differs using these two assembly methods (Fig. 2b). Typically, the reference-based method is highly sensitive in detecting low-abundance transcripts due to its ability to ‘fill in the gaps’ within transcripts, which is caused by low sequencing coverage [33]. In contrast, the de novo method usually creates a large number of incomplete transcripts when it comes to low coverage at certain regions. Thus, longer 3′-UTRs were expected from the reference-based assemblies. Compared to the other genomes analyzed, the bat genome was not as well assembled, and is relatively fragmented. That is probably the reason why no significant difference was observed in 3′-UTR lengths in the bat using these two methods.

**Fig. 5:**
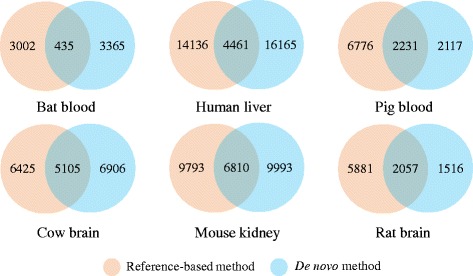
The overlap of 3′-UTRs predicted from both reference-based and de novo assemblies for each model species

The greatest merit of the *ExUTR* pipeline is its ability to predict 3′-UTR from massive transcripts without well-annotated reference genomes. To assess its application on de novo assemblies, we compared the whole set of 3′-UTR predicted from both reference-based and de novo methods for each species. We observed that a number of common 3′-UTRs were predicted by both reference-based and de novo methods while a proportion of unique 3′-UTRs were recovered by respective methods (Fig. 5). Particularly for bat, only 12% of 3′-UTRs were commonly detected by both methods. Most likely this resulted from the fact that closely-related *Myotis lucifugus* genome had to be used for the reference-based assembly of the *Myotis myotis* bat, as this is the phylogenetically closest whole genome available (See Additional file 1 for the method). For all other taxa both reference-based and de novo results were compared from the same species.

**Fig. 6:**
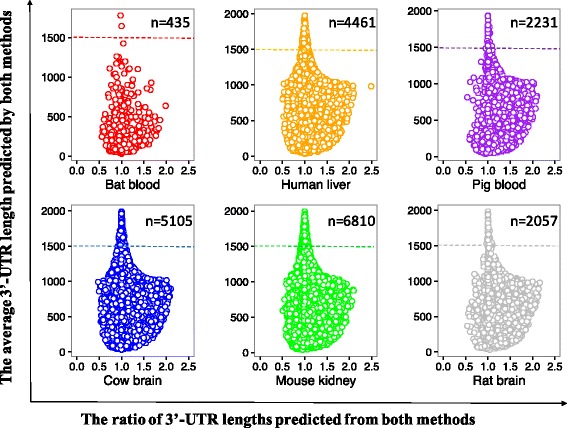
Comparisons of 3′-UTR lengths in the reference-based assembly and the de novo assembly for each species. 3′-UTR candidates predicted from both reference-based and de novo assemblies were used in the comparison where *n* indicates their number. The x-axis represents the ratios of 3′-UTR lengths predicted from the reference-based assembly to those from the de novo assembly. Prior to comparison, the lengths (bp) were log_2_-transformed. The y-axis represents the average length of each 3′-UTR predicted by two methods

For many pairs of 3′-UTR, the lengths predicted from the reference-based assemblies were slightly longer than those from the de novo assemblies, although in general, the differences were minor, with 82.9% ~ 94.3% of ratios of logarithmic 3′-UTR lengths from two assemblies falling between 0.5 and 1.5 for each species (Fig. 6). Interestingly, the common 3′-UTRs, whose lengths were above a rough threshold of 1500 bp, tended to exhibit high consistency in lengths predicted by both methods (Fig. 6). In addition, we also noticed that a number of unique 3′-UTR candidates were respectively recovered by each assembly method. These results indicate that if a well-assembled genome is not yet available for certain species, the de novo method can predict 3′-UTRs from RNA-Seq experiments using *ExUTR*, although the reference-based method is preferential due to the slightly longer 3′-UTR predicted.

### Validation of the *ExUTR* pipeline using 3P–Seq data

To assess the accuracy of the *ExUTR* pipeline, we analyzed four human cell line samples that have both RNA-Seq and corresponding polyA-position profiling by sequencing (3P–Seq) data publically available (See Additional file 1 for the method). From the analysis, on average 75.3% and 70.3% of the 3P–Seq reads were successfully mapped to the 3′-UTR candidates predicted from the reference-based and de novo RNA-Seq assemblies, respectively (Table 2). In addition, on average 93.0% and 82.1% of the 3′-UTR candidates predicted from the reference-based and de novo assemblies were recovered by the 3P–Seq data (Table 2). The high consistency observed between 3P–Seq and 3′-UTR prediction from RNA-Seq data highlights the accuracy of the *ExUTR* pipeline.

**Table 2:** Evaluation of *ExUTR* by using 3P–Seq data and corresponding RNA-Seq data

Cell line	Strategy	RNA-Seq	3P–Seq	
		Predicted 3′-UTR	Mapping rate	Percentage of 3′-UTR detected
HEK293	reference-based	18,935	76.8%	94.5%
	de novo	20,036	71.5%	82.0%
Hela	reference-based	17,412	78.3%	93.5%
	de novo	17,813	72.7%	80.8%
Huh7	reference-based	18,905	73.7%	89.0%
	de novo	19,950	69.1%	79.5%
IMR90	reference-based	14,962	72.2%	94.8%
	de novo	15,001	67.9%	86.1%

Predicted 3′-UTR: 3′-UTR candidates predicted from both reference-based and de novo assemblies using *ExUTR*; Mapping rate: the percentage of 3P–Seq reads mapped to the 3′-UTRs predicted from the corresponding RNA-Seq assembly; Percentage of 3′-UTR detected: the percentage of the predicted 3′-UTRs covered by 3P–Seq data

## Discussion

Regulatory regions within 3′-UTR are playing a pivotal role in mediating gene expression by providing both binding sites for regulatory proteins as well as miRNA. However, limited information of these molecular interactions is only available in a few model organisms, such as human, and such regulatory mechanisms tend to be species-specific. Therefore, genome-wide characterization of 3′-UTR sequences will remarkably facilitate our knowledge of the regulatory mechanisms of 3′-UTR, particularly for non-model organisms with limited genomic resources available. For this purpose, we developed *ExUTR*, an NGS-based pipeline that can predict and extract 3′-UTR sequences from massive RNA-Seq data in the absence of well-assembled and -annotated genomes.

According to the case studies, *ExUTR* successfully predicted and extracted a large number of 3′-UTR candidates from both model and non-model mammals, many of which have been well characterized (Figs. 2c and 3). Compared with model species where high-quality genomes are available, 3′-UTRs predicted from non-model species were significantly less well-characterized (Fig. 3). This may be largely due to lack of 3′-UTR resources of the related species. To assess the performance of *ExUTR* on de novo assembly which is the only option for non-model species, we compared the sets of 3′-UTR candidates predicted from model mammals using both reference-based and de novo strategies. Aside from the common 3′-UTRs, a number of unique 3′-UTRs were recovered by respective methods. This could be explained by the fact that the reference-based method enables the detection of lowly expressed 3′-UTRs with high sensitivity whereas the de novo method can recover exogenous 3′-UTRs that are missing in the genome.

Although a large number of 3′-UTRs predicted through the *ExUTR* pipeline were well characterized based on the curated 3′-UTR resources, we further validated the accuracy of *ExUTR* without the information of genomes or well-defined protein sequences by employing 3P–Seq data. The analyses of human cell line RNA-Seq and corresponding 3P–Seq data indicate that a large proportion of 3′-UTRs predicted from both reference-based and de novo method through *ExUTR* were covered by 3P–Seq data (Table 2). The high 3P–Seq mapping rates and 3′-UTR coverage by 3P–Seq (Table 2) imply that most of 3′-UTRs predicted through *ExUTR* that currently have not been characterized are the genuine 3′-UTRs. This blind validation method demonstrates the accuracy of the *ExUTR* pipeline regardless of the availability of reference genomes or related 3′-UTR resources.

Although *ExUTR* enables a genome-wide prediction of 3′-UTR sequences from massive RNA-Seq data without well-assembled and -annotated genomes, it does depend on genomic resources, particularly well-defined protein sequences. Therefore, the performance of *ExUTR* relies largely on the availability of the curated protein resources. In this study, we tested the *ExUTR* pipeline by using both model and non-model mammalian species, and *ExUTR* exhibited great potential to predict and extract 3′-UTRs. However, *ExUTR* may fail to predict 3′-UTRs on a genome-wide scale when being applied to poorly-studied species with a scarcity of relevant genomic or protein information available. Under this circumstance, 3P–Seq could be an alternative strategy to characterize 3′-UTRs in these species. In addition, alternative polyadenylation has recently gained considerable attention as it is recognized as a major mechanism of gene regulation [34]. However, *ExUTR* only takes well-assembled transcripts as inputs and outputs corresponding predicted 3′-UTR candidates, but cannot automatically report alternative 3′-UTR sequences. Tools, such as CD-HIT [35], could be used to categorize alternative 3′-UTRs based on the 3′-UTR candidates predicted through *ExUTR*.

### Computational performance

*ExUTR* was designed to allow genome-wide prediction of 3′-UTR sequences from RNA-Seq data on a standard desktop within a reasonable amount of time. The largest contribution to runtime would be the step of sequence alignment using BLAST. Excluding transcriptome assembly, which is optional, runtime hinges on the number of transcripts, transcriptome complexity and CPU numbers. For instance, on the desktop computer whose configuration was aforementioned, it took ~10.75 h to predict 3′-UTR from 186,053 transcripts of human liver while only ~2.25 h was needed for 22,265 transcripts from bat blood (Table 1). There is no significance difference in the runtime between two assemblies of human liver, although the de novo method generated 3 times more transcripts than the reference-based method (Table 1). This can be explained by the low complexity of the de novo assembly which contained many short, incomplete or non-coding transcripts without potential ORFs that required less time for BLAST to process. Since BLAST supports multi-threads tasks, runtime could be reduced with increased CPU resources, and the overall process has minimal memory requirement.

### Comparison with other 3′-UTR prediction tools

To compare the performance of one pipeline with another, the best approach is to have the same data analyzed by multiple pipelines and compare the results. However, due to the difference of throughput capabilities and the availability of genome annotation, we could not fully compare the *ExUTR* pipeline with current 3′-UTR prediction tools using our data. Where possible, we compared some features between the *ExUTR* pipeline and other extant tools (Table 3). Unlike 3USS and UTRscan which are web-based tools with user-friendly interfaces, *ExUTR* is implemented as standalone and enables the high-throughput prediction of 3′-UTR sequences from RNA-Seq data. Despite handling large-scale data, minimal computational resources are required when compared with GETUTR which usually demands a huge amount of memory. This efficient computation allows the performance of the whole process on a standard desktop with reasonable runtime. More importantly, the implementation of *ExUTR* is based mainly on the intrinsic signals of transcripts, which is especially useful in scenarios where reference genomes or annotations are missing. This particularly facilitates the identification of 3′-UTR from massive RNA-Seq experiments of non-model organisms, leading to a better understanding of 3′-UTR biology.

**Table 3:** Comparisons between *ExUTR* and other 3′-UTR mining tools

	ExUTR	GETUTR	3USS	UTRscan
Single transcript	Yes	No	Yes	Yes
De novo assembly (genome-independent)	Yes	No	No	No
Reference-based assembly	Yes	Yes	No	No
High-throughput	Yes	Yes	No	No
Web-based, user-friendly interface	No	No	Yes	Yes
Computational resources requirement	Low	High	–	–

## Conclusions

To address the demands for genome-wide prediction of 3′-UTR sequences from NGS data, we developed *ExUTR*, an automated pipeline that is able to handle massive RNA-Seq data without well-annotated genomes and with minimal cost of computational resources. The success of its application on both model and non-model species demonstrates the functionality of the pipeline, and analyses of RNA-Seq and their corresponding 3P–Seq data reveal the accuracy of *ExUTR*. With its broad range of application, *ExUTR* could be a powerful tool to predict and retrieve 3′-UTR from RNA-Seq experiments of countless species for which RNA-Seq is available, thereby leading to the better understandings of post-transcriptional regulation, 3′-UTR evolution and the mechanisms of miRNA-mRNA interaction.

## Availability and requirements

**Project name:** ExUTR.

**Project home page:** https://github.com/huangzixia/ExUTR

**Operating system(s):** Linux.

**Programming language:** Perl & Bash.

**Other requirements:** BioPerl module.

**License:** GNU GPLv2.

**Any restrictions to use by non-academics:** None.

## Additional files

Additional file 1: The detailed methods for the analyses of the demonstrated RNA-Seq data. (DOCX 149 kb)

## Abbreviations

3′-UTR: Three prime untranslated region
3P–Seq: polyA-position profiling by sequencing
AREs: AU-rich elements
FPKM: Fragments per kilobase per million
MREs: miRNA response elements
NGS: Next generation sequencing
PASs: Polyadenylation signals
