# Supplementary material for: ExUTR: a novel pipeline for large-scale prediction of 3′-UTR sequences from NGS data
Source: BMC Genomics. 2017 Nov 6;18:847. doi: 10.1186/s12864-017-4241-1 (PMC5674806; doi:10.1186/s12864-017-4241-1)
Supplement: Supplementary file 1 — The detailed methods for the analyses of the demonstrated RNA-Seq data. (DOCX 149 kb) [file 12864_2017_4241_MOESM1_ESM.docx]

**Supplementary Text S1**

**Materials and Methods**

*Data for demonstration*

To demonstrate the functionality of the *ExUTR* pipeline, we obtained publically available RNA-Seq data from the NCBI SRA database. These RNA-Seq data covers six model mammalian species and six non-model mammals across diverse tissue types. See **Table 1** for SRA accession numbers. In addition, to evaluate the accuracy of *ExUTR*, four human cell line 3P-Seq and their corresponding RNA-Seq data were also employed. See SRA accession numbers below.

*Transcriptome assembly*

Initially, the quality of the raw data was inspected and screened for each sample. Adaptors and low quality bases (less than Q20) were trimmed using cutadapt (version 1.7.1) [[1](#_ENREF_1)]. The resulting reads, whose lengths were longer than 50 bp, were further retained for transcriptome assembly. We used both reference-based and *de novo* methods to assemble transcriptome for each species. For reference-based method, the genome sequences of each species were downloaded, and further indexed using bowtie2 (version 2.1.1) [[2](#_ENREF_2)]. As the bat (*Myotis myotis*) genome is currently not available, we used the genome of its closely-related species, *Myotis lucifugus*, as a compromise. The post-processed reads were mapped to the genome references using Tophat2 (version 2.0.11) [[3](#_ENREF_3)], and further assembled into transcripts by Cufflinks (version 1.3.0) [[4](#_ENREF_4)]. Since Cufflinks automatically reports FPKM value for each transcript, the final set of assembled transcripts was determined by removing the lowly-expressed transcripts (FPKM < 0.1). For *de novo* method, the post-processed reads were assembled into transcripts using Trinity (version 20140717) [[5](#_ENREF_5)]. Since the assembly contained some highly similar transcripts, the redundancy of these transcripts was removed by CD-HIT [[6](#_ENREF_6)] with an identity cutoff of 95%, and only the longest transcript from each redundant cluster was kept. Followed by the redundancy removal, transcripts were quantified using Sailfish (version 0.7) [[7](#_ENREF_7)]. We excluded the lowly-expressed transcripts (TPM < 0.1) from further analyses. The default parameter settings were applied for all tools.

*3’-UTR prediction and analyses*

For each model species, 3’-UTRs were predicted and retrieved from the reference-based and *de novo* assemblies, respectively, through the *ExUTR* pipeline whereas for each non-model species 3’-UTRs were only predicted using the *de novo* method. The completeness of transcriptome was assessed for each assembly using Core Eukaryotic Genes Mapping Approaches (CEGMA) [[8](#_ENREF_8)]. For the species with well-annotated genomes available, the predicted 3’-UTRs were annotated and validated by comparing them to the characterized 3’-UTR sequences obtained from their respective genomes through Ensembl, using BLASTN [[9](#_ENREF_9)] with the E-value cutoff of 10^-10^, while for the species lack of high-quality genomes, the predicted 3’-UTRs were annotated and validated by the UTRdb database [[10](#_ENREF_10)] using BLASTN [[9](#_ENREF_9)] with the same parameter settings. Subsequently, student’s t-tests were carried out to compare the length distributions of 3’-UTRs predicted from both assemblies for each species, and P-value < 0.01 was deemed significant. To obtain the common 3'-UTR predicted from both assemblies for each species, we compared their whole sets of 3’-UTR using reciprocal BLASTN [[9](#_ENREF_9)], with the E-value cutoff of 10^-10^. Only 3’-UTRs, which showed reciprocal hit to each other from both two methods, were considered as common 3’-UTRs.

*Validation of ExUTR using 3P-Seq data*

Four human cell line samples (HEK293, Hela, Hub7 and IMR90) that have both 3P-Seq data and corresponding RNA-Seq data were used to assess the accuracy of the *ExUTR* pipeline. The reference-based and *de novo* assemblies of each RNA-Seq sample were assembled as described above. The 3’-UTR candidates were predicted from these assemblies using *ExUTR*. The 3P-Seq data were subsequently mapped to these predicted 3’-UTRs correspondingly using sailfish [[7](#_ENREF_7)] (kmer length: 15), and the 3P-Seq mapping rates and the percentage of 3’-UTRs that were covered by the 3P-Seq data were further investigated. The accession numbers used in this analysis are SRR1033823, SRR1033822, SRR1033821 and SRR1033820 for 3P-Seq, and SRR1032895, SRR1032890, SRR1032883 and SRR1032877 for RNA-Seq.

**References**

1. Martin M: **Cutadapt removes adapter sequences from high-throughput sequencing reads**. *EMBnet journal* 2011, **17**(1):pp. 10-12.

2. Langmead B, Salzberg SL: **Fast gapped-read alignment with Bowtie 2**. *Nature methods* 2012, **9**(4):357-359.

3. Kim D, Pertea G, Trapnell C, Pimentel H, Kelley R, Salzberg SL: **TopHat2: accurate alignment of transcriptomes in the presence of insertions, deletions and gene fusions**. *Genome biology* 2013, **14**(4):R36.

4. Ghosh S, Chan CK: **Analysis of RNA-Seq Data Using TopHat and Cufflinks**. *Methods in molecular biology* 2016, **1374**:339-361.

5. Haas BJ, Papanicolaou A, Yassour M, Grabherr M, Blood PD, Bowden J, Couger MB, Eccles D, Li B, Lieber M *et al*: **De novo transcript sequence reconstruction from RNA-seq using the Trinity platform for reference generation and analysis**. *Nature protocols* 2013, **8**(8):1494-1512.

6. Li W, Godzik A: **Cd-hit: a fast program for clustering and comparing large sets of protein or nucleotide sequences**. *Bioinformatics* 2006, **22**(13):1658-1659.

7. Patro R, Mount SM, Kingsford C: **Sailfish enables alignment-free isoform quantification from RNA-seq reads using lightweight algorithms**. *Nature biotechnology* 2014, **32**(5):462-464.

8. Parra G, Bradnam K, Korf I: **CEGMA: a pipeline to accurately annotate core genes in eukaryotic genomes**. *Bioinformatics* 2007, **23**(9):1061-1067.

9. Altschul SF, Gish W, Miller W, Myers EW, Lipman DJ: **Basic local alignment search tool**. *Journal of molecular biology* 1990, **215**(3):403-410.

10. Grillo G, Turi A, Licciulli F, Mignone F, Liuni S, Banfi S, Gennarino VA, Horner DS, Pavesi G, Picardi E *et al*: **UTRdb and UTRsite (RELEASE 2010): a collection of sequences and regulatory motifs of the untranslated regions of eukaryotic mRNAs**. *Nucleic acids research* 2010, **38**(Database issue):D75-80.
